# A Putative Frizzled 7-Targeting Compound Acts as a Firefly Luciferase Inhibitor

**DOI:** 10.1021/acs.jmedchem.4c02766

**Published:** 2024-12-13

**Authors:** Julia Kinsolving, Lukas Grätz, Jan Hendrik Voss, Bente Löw, Emily Shorter, Baptiste Jude, Johanna T Lanner, Stefan Löber, Peter Gmeiner, Gunnar Schulte

**Affiliations:** 1 Section of Receptor Biology & Signaling, Dept. Physiology & Pharmacology, Karolinska Institutet, Stockholm S-171 77, Sweden; 2 Department of Chemistry and Pharmacy, Friedrich-Alexander-Universität, Erlangen 91058, Germany; ^3^Molecular Muscle Physiology and Pathophysiology, Dept. Physiology & Pharmacology,^4^Division of Pediatric Neurology, Dept of Women's and Children's Health Karolinska Institutet, Stockholm S-171 77, Sweden

## Abstract

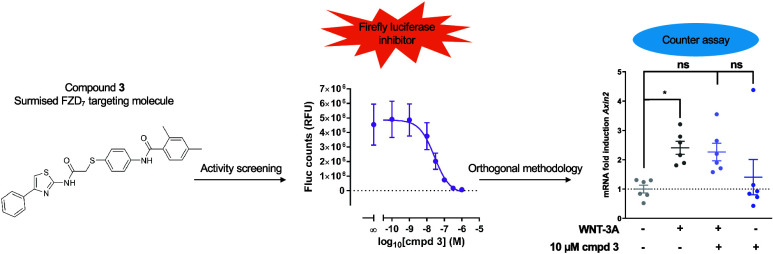

The Frizzled family (FZD_1–10_) of G protein-coupled receptors regulates WNT signaling mediating proliferative input. Dysregulation of FZD_7_ and exaggerated WNT/β-catenin signaling is frequently observed in intestinal cancers. Therefore, it is attractive to develop therapeutics targeting FZD_7_ for cancer treatment. Structure-based virtual screening has identified compound 28, which inhibited WNT/β-catenin signaling based on the luciferase-based reporter gene TOPFlash assay. However, upon pharmacological validation, compound 28 rather acts as a potent Firefly luciferase (Fluc) inhibitor (IC_50_ = 30 nM), matching the reported IC_50_ for compound 28-mediated inhibition in the TOPFlash assay. Moreover, we employed Fluc-independent assays, a FZD_7_-focused bioluminescence resonance energy transfer biosensor and quantitative PCR, to emphasize the inability of compound 28 to inhibit the WNT-3A-induced conformational dynamics in FZD_7_ and transcription of *Axin2,* a WNT target gene. Thus, we underline the importance of counter screens to validate compounds that interfere with the detection technology used for compound screening.

## Introduction

The Frizzled (FZD) family of G protein-coupled receptors (GPCRs) consists of ten paralogs (FZD_1–10_), which are the receptors for the Wingless/Int1 (WNT) family of lipoglycoproteins.^1,2^ WNT/FZD signaling is essential during embryonic development and balanced signaling input is a prerequisite for adult tissue homeostasis for example in regenerative epithelium.^3^ Consequently, upregulated WNT/FZD signaling can result in exaggerated proliferative input manifesting in diverse forms of cancer rendering FZDs an attractive target for drug discovery approaches. While the recent years have been dominated by the development of FZD-targeting biologics, such as FZD-binding antibodies, WNT-derived peptides, miniWNTs, FZD-targeting peptides, and bispecific WNT-mimetics (so-called WNT surrogates),^4−8^ increasing structural information about FZDs opens new opportunities to find FZD-targeting small molecule compounds in a structure-guided manner.^9−14^ So far very few purely experimental FZD-targeting small molecules with diverse modes of action have emerged. For example, small molecules were reported to target the orthosteric WNT-binding site on the extracellular cysteine-rich domain (CRD)^15^ as well as intracellular, allosteric sites.^16,17^ Furthermore, partial agonists^18^ and negative allosteric modulators^19^ addressing the central cavity within the seven transmembrane (7TM) core of FZDs have been identified. Moreover, the recent high-resolution inactive structure of FZD_7_ has allowed for an in-depth analysis of activation mechanisms and an allosteric cholesterol molecule that could potentially be targeted by small molecules.^14^ Generally, the quest for FZD-targeting small molecule compounds has been hampered by a lack of understanding of basic FZD pharmacology including details of receptor activation, as well by a lack of screening methodology reporting directly on FZD activation rather than on downstream signaling events such as gene transcription.^20,21^

Of the ten family members, FZD_7_ is upregulated in multiple forms of cancer and is associated with cancer cell proliferation and tumor development. In this context, FZD_7_ is particularly relevant in intestinal cancer,^22−25^ breast cancer,^26−28^ and ovarian cancer.^29^ The considerable interest toward FZD_7_ due to its role in cancer in combination with a recent increase in structural information for FZDs has put rational drug design of FZD_7_ inhibitors at the forefront.^5^ In silico docking campaigns have resulted in compounds with apparent biological activity. One example includes the discovery of small molecules, SRI-37892 and SRI-35959, that share a phenyl benzimidazole moiety that can be docked to the 7TM domain of FZD_7_ and inhibit WNT/β-catenin signaling in WNT-3- and LRP6-expressing HEK293 cells.^19^ Furthermore, these compounds were shown to compromise the growth of selected tumor cell lines, even though the claimed biological antitumor effects were not controlled for cell toxicity. The molecules were identified from a structure-based virtual screen using a FZD_7_ homology model that was based on a crystal structure of Smoothened (SMO) in complex with an antagonist.

**Figure 1:**
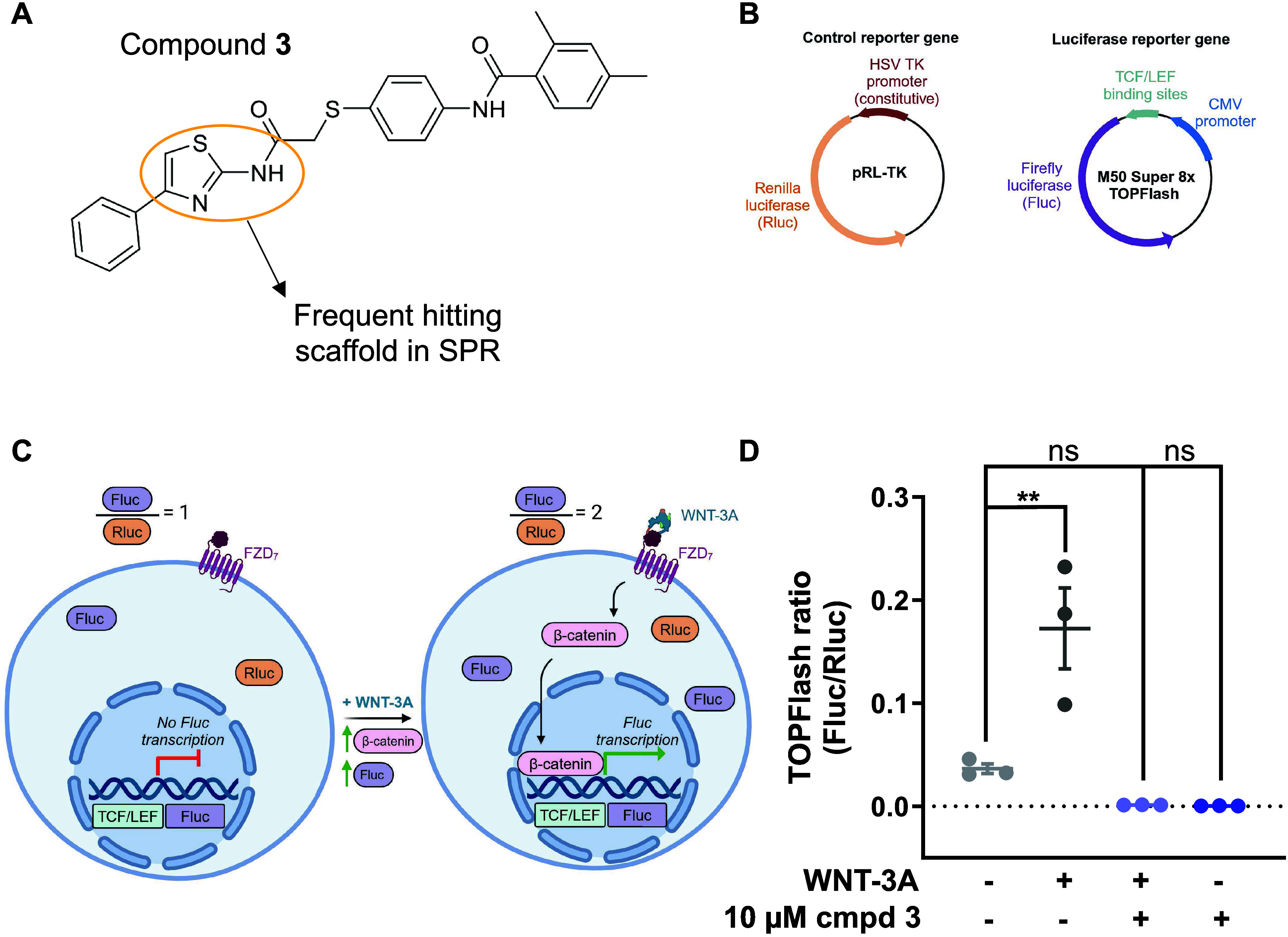
Compound **3** abrogates the basal and WNT-3A-induced TOPFlash signal. (A) Chemical structure of compound **3** (corresponding to compound 28). The 2-aminothiazole group is circled in orange. (B) Two plasmids, Renilla luciferase (Rluc) and M50 Super 8x TOPFlash (Fluc), are used in combination to transfect ΔFZD_1–10_ HEK293T cells. (C) Schematic of the TOPFlash assay used to quantify WNT/β-catenin pathway activation induced by WNT-3A-induced activation of HiBiT-FZD_7_. (D) Reported TOPFlash ratio response in ΔFZD_1–10_ HEK293T cells transfected with HiBiT-FZD_7_. 10 μM compound **3** was added and subsequently WNT-3A (300 ng/mL) or vehicle and incubated for 24 h. Data are presented as means ± SEM of three biological replicates (**p < 0.01, n.s. not significant) (one-way ANOVA with Dunnett’s multiple comparisons test). Parts of this figure were created with BioRender.com released under a Creative Commons Attribution-NonCommerical-NoDrivs 4.0 International license.

Similarly, another set of small molecules based on a different scaffold was identified by structure-based virtual screening using the FZD_7_-G_s_ cryo-EM structure as a starting point.^11^ In silico docking of a large compound library resulted in FZD_7_-targeting hits, which were synthesized and followed up using a structure–activity relationship (SAR) campaign. The SAR-driving assay was based on the widely used concept of a bioluminescence-based reporter gene assay called TOPFlash,^30^ which relies on a ratiometric readout derived from the bioluminescence of Firefly and Renilla luciferases in the cell lysates. The SAR resulted in a hit optimization leading to a most prominent compound, compound 28, with a reported IC_50_ of approximately 30 nM in HEK293T cells transfected with the TOPFlash reporter^11^ (Figure 1A). Reporter gene assays using luciferase allow for high-throughput screening campaigns. Interestingly, several false positive hits have emerged from previous GPCR-focused luciferase reporter-gene assay screens due to compound-mediated luciferase inhibition.^31^

The interaction of the compound with FZD_7_ was supposedly supported using surface plasmon resonance (SPR). Compound 28 resulted in a concentration-dependent increase in the SPR signal suggesting direct interaction with solubilized and purified FZD_7_ but not SMO. The SPR data were interpreted as a selectivity for compound 28 with respect to class F GPCRs, specifically FZD_7_. It should, however, be mentioned that the SPR experiments did not include any experimental paradigm (competitive ligand, nonbinding ligand, mutagenesis) that would support specific binding site engagement of the compound on FZD_7_. Importantly, the TOPFlash assay-based SAR campaign did not include a counter assay to control for the risk of interference of compound 28 or other tested compounds with the assay methodology.^32^ In this context it is also important to note that the molecular scaffold of compound 28 belongs to the compound group of 2-aminothiazoles, so-called promiscuous 2-aminothiazoles or PrATs, which are frequently interfering with biophysical binding assays^33^ (Figure 1A). Moreover, thiazoles have been previously described as problematic scaffolds in respect to Fluc inhibition.^34^

Here, we aimed to thoroughly validate compound 28 as a putative FZD_7_-targeting small molecule. Assessment of the ability of compound 28 to inhibit basal and WNT-3A-induced FZD signaling based on the broadly used TOPFlash assay underlined that this small molecule completely abrogates the basal and WNT-induced TOPFlash signal. While compound interference is widely reported due to off-target activity such as luciferase inhibition, counter-screens are routinely employed in parallel to the primary screen to assess potential interference.^34^ The creation of a suitable counter assay to control for assay interference by means of expressing a constitutively expressed Firefly luciferase (Fluc) in FZD_1–10_ knockout cell lines (ΔFZD_1–10_ HEK293T) cells indicated, however, that compound 28 abrogated Fluc (but not Rluc or Nluc) bioluminescence rather than targeting FZDs as a pharmacologically relevant target. Moreover, to further probe the possible molecular mechanism of compound 28 on WNT-signaling, we employed a genetically encoded bioluminescence resonance-energy transfer (BRET)-based biosensor called FZD_7_-DEP-Clamp^35^ and quantitative PCR (qPCR) to detect WNT-3A-induced conformational rearrangements in FZD_7_ and target gene transcription, respectively. Our results demonstrate that compound 28 had no effect on WNT-3A-induced changes reported by the FZD_7_-DEP-Clamp or gene regulation underlining that compound 28 does not act at FZD_7_ or targets the WNT/β-catenin signaling pathway, as was reported. Most importantly, our work emphasizes the relevance and importance of suitable counter assays for validation of small molecules not only during the discovery of FZD-targeting compounds but in all screening-based drug discovery efforts.

## Results

### Synthesis of Compound **3**

The report of compound 28 as a FZD-targeting small molecule motivated us to synthesize this molecule for validation of the compound’s mode of action and for potential structural investigation using cryo-EM. For these purposes, we synthesized compound 28 according to previously published protocols including stepwise synthesis of 2-chloro-*N*-(4-phenylthiazol-2-yl)acetamide (compound **1**), 2-((4-aminophenyl)thio)-*N*-(4-phenylthiazol-2-yl)acetamide (compound **2**), and 2,4-dimethyl-*N*-(4-((2-oxo-2-((4-phenylthiazol-2-yl)amino)ethyl)thio)phenyl)benzamide herein referred to as compound **3** (Figure S1). The integrity and purity of compound **3** were validated using HPLC and ^1^H NMR with a reported purity of >95% (Figure S2).

### Compound **3** Abrogates Basal and WNT-3A-Induced TOPFlash

To quantify the effect of compound **3** (compound 28; Figure 1A) on WNT/β-catenin signaling exclusively mediated by FZD_7_, the TOPFlash luciferase reporter assay was conducted in ΔFZD_1–10_ HEK293T cells transiently transfected with HiBiT-FZD_7_. These cells are engineered by CRISPR/Cas9 to create a cellular system completely devoid of endogenous FZDs and the possibility to retransfect individual FZDs for assessment of WNT-induced signaling in a paralog-selective manner.^36^ The TOPFlash assay is based on two distinct luciferases, Firely luciferase (Fluc, from the Firefly *Photinus pyralis*) and Renilla luciferase (Rluc, from the sea pansy *Renilla reniformis*). The light emissions from these two bioluminescent proteins are used in a ratiometric readout for quantifying the transcriptional activity along β-catenin-dependent signaling cascades. The TOPFlash plasmid (M50 Super 8x TOPFlash) contains 7 concatenated T-cell factor/lymphoid enhancer binding factor (TCF/LEF) binding sites, which regulate the transcription of the gene encoding Firefly luciferase. The Rluc construct, pRL-TK, provides constitutive Renilla luciferase expression serving as an internal control to normalize for transfection efficiency (Figure 1B). An increase in WNT/β-catenin signaling activity leads to increased Fluc expression and subsequent bioluminescence (Figure 1C). In the cellular system of ΔFZD_1–10_ HEK293T cells transfected with human HiBiT-FZD_7_, WNT-3A (300 ng/mL) elicited a substantial increase in the TOPFlash ratio. Compound **3** abrogated both the basal (no WNT-3A) and the WNT-3A-induced TOPFlash signal below basal levels (Figure 1D) in full agreement with the previous report.^11^ The experiments were performed in the presence of the porcupine inhibitor C59 to prevent the contribution of endogenously produced and secreted WNTs. Thus, we confirmed that the newly synthesized compound **3** reproduced previously reported data on WNT-induced WNT/β-catenin signaling and the effect of compound 28 thereon.

### Compound **3** Is a Potent Luciferase Inhibitor

While the luciferase Fluc is widely employed in molecular biology, this use comes with caveats of potential luciferase inhibition and assay interference.^37^ For example, a small molecule library available in PubChem showed 12% of the compounds acted as Firefly luciferase inhibitors based on concentration response curves (CRCs).^38^ Furthermore, a screening program at Novartis revealed a hit rate of more than 30% of potential Fluc inhibitors with nanomolar potency in their groups of compounds, which presumably docks in the Fluc active site.^32^

**Figure 2:**
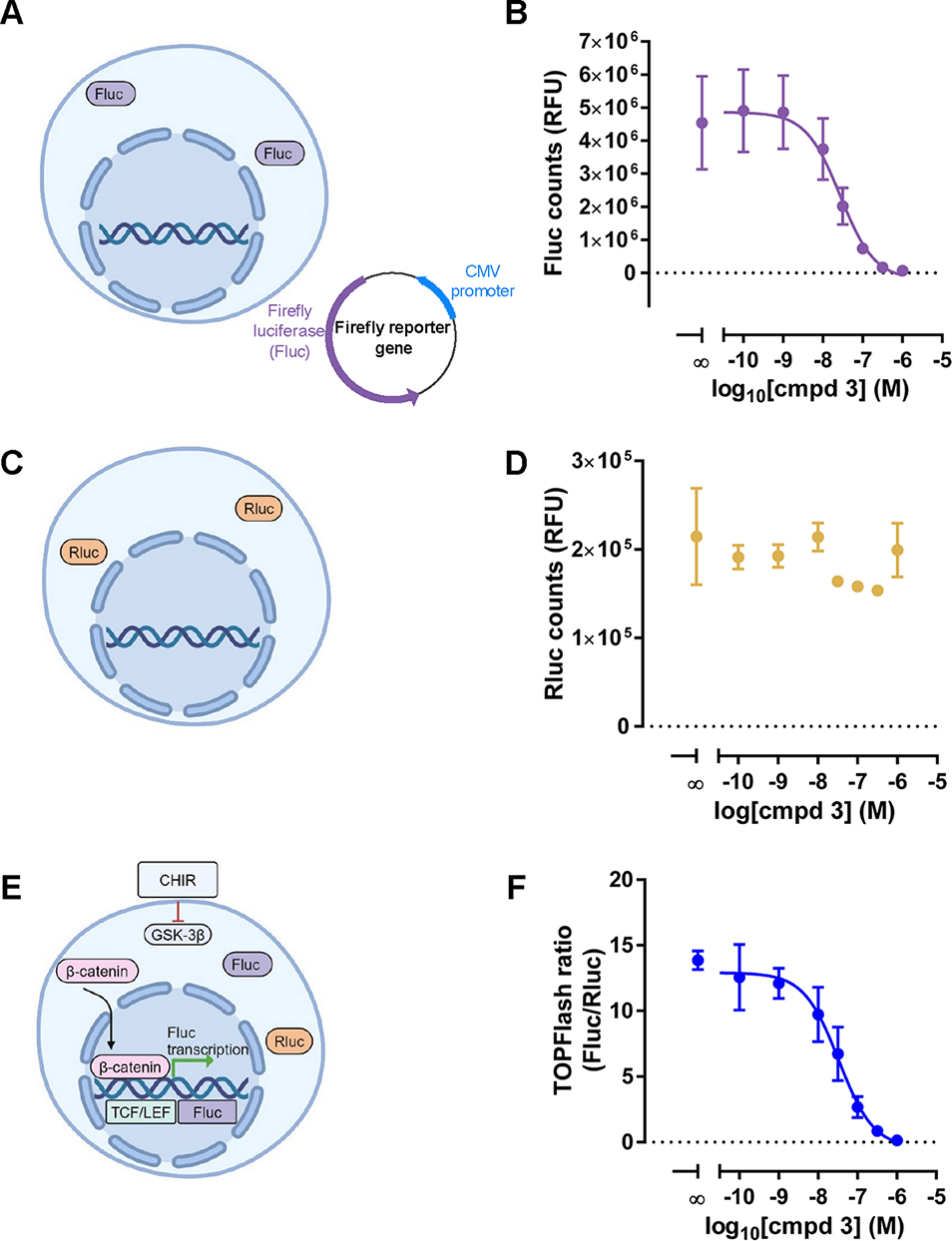
Compound **3** is a potent Firefly luciferase inhibitor. (A) Schematic of the counter assay performed in ΔFZD_1–10_ HEK293T cells where Fluc was transiently transfected in the absence of WNTs or FZDs driven solely by a CMV promoter. (B) A concentration–response curve highlights the effect of increasing concentrations of compound **3** on luminescence emerging from soluble Fluc. Data are shown as raw luminescence counts of three biological replicates with means ± SEM. IC_50_ = 25 nM (pIC_50_ 7.625 ± 0.02) (C) Schematic of the counter assay performed in ΔFZD_1–10_ HEK293T cells where Rluc was transiently transfected in the absence of WNTs or FZDs. (D) Concentration–response curve shows the lack of an effect of increasing concentrations of compound **3** on luminescence emerging from soluble Rluc. Data are are shown as raw luminescence counts of three biological replicates with means ± SEM from three biological replicates. (E) Schematic of the counter assay in ΔFZD_1–10_ HEK293T cells where Fluc and Rluc were transfected in the presence of 10 μM CHIR99021. (F) Concentration–response curve demonstrating the inhibitory action of compound **3** on TOPFlash activity in the presence of 10 μM CHIR99021. Data are presented as TOPFlash ratios (Fluc/Rluc) with means ± SEM of three biological replicates. IC_50_ is 33 nM (pIC_50_ of 7.47 ± 0.08). All conditions contain 10 nM C59. Parts of this figure were created with BioRender.com released under a Creative Commons Attribution-NonCommerical-NoDrivs 4.0 International license.

Thus, to investigate a potentially signaling-independent effect of compound **3** on Fluc bioluminescence, we performed a counter experiment, where Fluc expression was driven by a constitutive cytomegalovirus (CMV) promotor, instead of being regulated by WNT/β-catenin pathway activation (Figure 2A). The Fluc bioluminescence was then assessed in the presence of increasing concentrations of compound **3** in ΔFZD_1–10_ HEK293T cells barring any FZDs. Also, C59-mediated porcupine inhibition secured that endogenously secreted WNTs could not contribute to any signaling. Compound **3** reduced Fluc bioluminescence in a concentration-dependent manner resulting in a sigmoidal and monophasic concentration response curve (Figure 2B). Thus, we conclude that compound **3** completely inhibits Fluc luciferase activity with an IC_50_ of 25 nM (pIC_50_ of 7.625 ± 0.015, reported as SEM), matching the reported IC_50_ for inhibition of WNT/β-catenin signaling.^11^

Given that TOPFlash is a ratiometric readout between Fluc and Rluc, we sought to investigate the effects of compound **3** also on Rluc. Therefore, we aimed to assess the effect of compound **3** solely on Rluc bioluminescence in the absence of FZDs or WNT input (Figure 2C). In this assay set up, Rluc bioluminescence was not affected by increasing concentrations of compound **3** (Figure 2D) strengthening the hypothesis that compound **3** acts specifically on Fluc.

With the findings that compound **3** acts solely on Fluc completely independent of FZDs and WNT and consequently abrogates the TOPFlash response, we sought to investigate compound effects on the TOPFlash signal when signaling is stimulated by addition of a small molecule GSK-3β inhibitor, CHIR99021. Treatment with CHIR99021 is commonly used to initiate receptor-independent β-catenin signaling by inhibiting GSK-3β for example in HEK293 cells at a concentration of 10 μM.^39^ To examine the effect of compound **3** on a CHIR99021-induced TOPFlash signal, we used increasing amounts of compound **3** in TOPFlash-transfected ΔFZD_1–10_ HEK293T cells treated with 10 μM CHIR99021 overnight (Figure 2E). Similar to the reported IC_50_ for luciferase inhibition, compound **3** inhibited the CHIR99021-induced TOPFlash ratio with an IC_50_ of 33 nM (pIC_50_ of 7.47 ± 0.076, reported as SEM) (Figure 2F) despite the complete absence of FZDs, further corroborating the FZD-independent effect of compound **3** on the TOPFlash readout.

### WNT-Induced Conformational Dynamics and Target Genes Are Unaffected by Compound **3**

**Figure 3:**
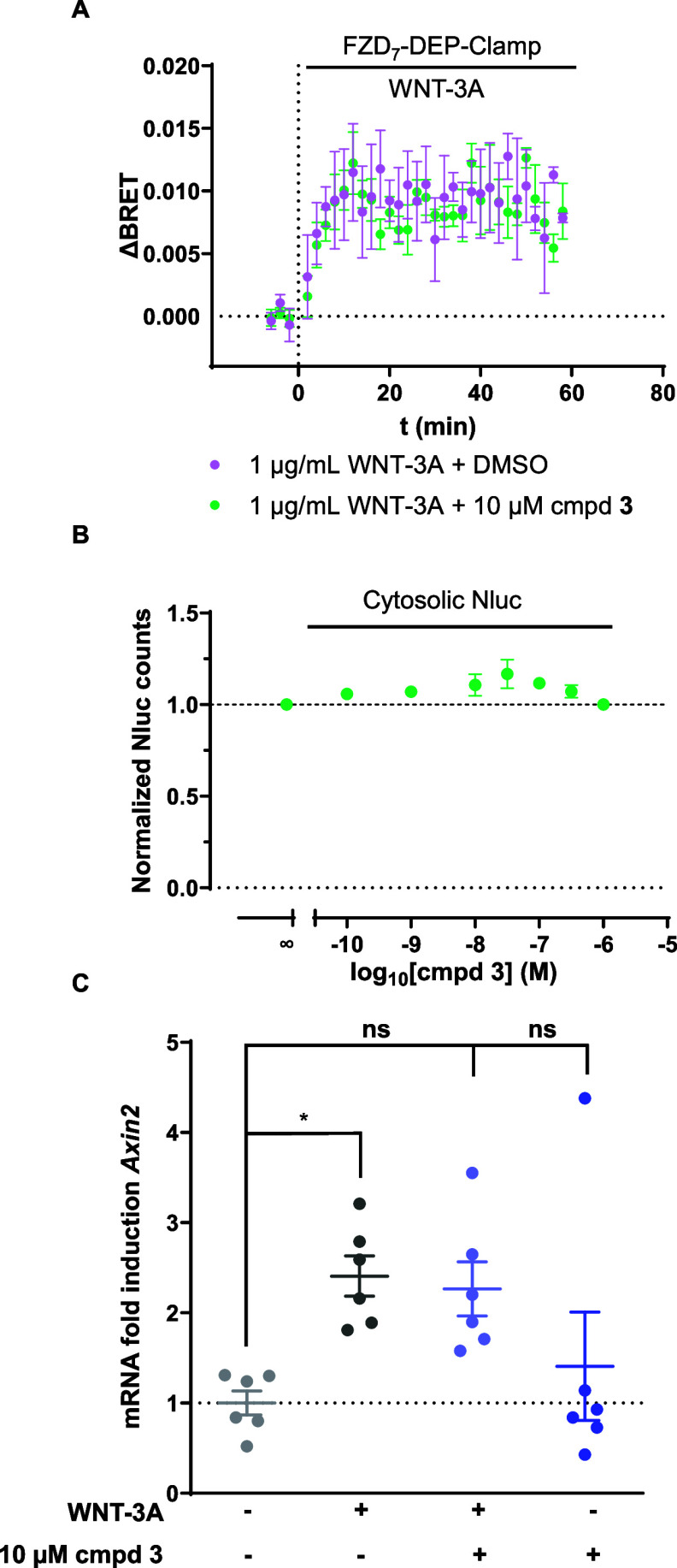
Orthogonal assays depict the lack of effect of compound **3** on the WNT-induced conformational rearrangements or bona fide WNT target gene, *Axin2.* (A) The kinetic profile of the unimolecular FZD_7_-DEP sensor upon WNT-3A stimulation and DMSO (purple) vs WNT-3A stimulation with compound **3** (green) are shown. Experiments were conducted in ΔFZD_1–10_ HEK293T cells transiently transfected with FZD_7_-DEP-Clamp. Data are presented as ΔBRET ratios from three independent experiments with means ± SEM. (B) A concentration response curve depicts the effect of increasing concentrations of compound **3** on cytosolic Nluc (TK-Nluc). Data are presented as normalized Nluc counts to conditions in the absence of compound 3 (=1) with means ± SEM of three biological replicates. (C) Mouse embryonic fibroblasts (MEFs) were kept unstimulated or stimulated with either 200 ng/mL WNT-3A, or 10 μM compound **3** in the presence of 10 nM C59 for 24 h. Afterward, cells were analyzed for expression *Axin2* mRNA by quantitative PCR (qPCR). Data is reported as fold-increase relative to baseline conditions (serum-free medium with C59) and log2 transformed. Error bars indicate mean ± SEM of six biological replicates (*p < 0.05, n.s. not statistically significant; one-way ANOVA with Dunnett’s multiple comparisons test).

Even though our results suggest that compound **3** is a potent Fluc inhibitor and thus interferes with the Fluc-based TOPFlash readout, the possibility remains that it is indeed active and capable of inhibiting WNT/β-catenin signaling by targeting FZD_7_. In order to employ a FZD_7_-focused read out, we transfected ΔFZD_1–10_ HEK293T cells with a recently reported BRET-based biosensor called FZD_7_-DEP-Clamp.^35^ WNT-3A elicits an increase in BRET using the FZD_7_-DEP-Clamp reporting on agonist-induced conformational rearrangements in the FZD-DVL interface.^40^ A FZD-targeting negative allosteric modulator (NAM) should interfere with the WNT-induced increase in the FZD_7_-DEP-Clamp signal.However, 10 μM compound **3** did not affect the FZD_7_-DEP-Clamp signal elicited by WNT-3A (Figure 3A). In this experimental paradigm, it must be underlined that a positive control in the form of another FZD-targeting NAM is currently not available. In order to make use of the FZD_7_-DEP-Clamp sensor, we also validated the effect of compound **3** on cytosolic Nluc. Obviously, an inhibitory effect of compound **3** on Nluc would have provided false results in combination with the Nluc-dependent BRET biosensor. Expression of Nluc under the control of a TK promotor in ΔFZD_1–10_ HEK293T cells and Nluc bioluminescence recording in the presence of vehicle or increasing concentrations of compound **3** revealed no effect of compound 3 on luciferase output (Figure 3B).

Another hallmark of WNT-3A stimulation is the increase in the transcription of WNT-target genes such as *Axin2*.^41^ Evaluation of *Axin2* mRNA by quantitative PCR (qPCR) allows for a sensitive assay to measure gene expression levels in samples treated with WNTs or other compounds. This approach was for example used to quantify the expression of WNT target genes in blastula embryos at different time points of development,^42^ the effect of the porcupine-inhibitor IWP-2 on transcriptional activity of WNT genes in breast cancer cell lines,^43^ and the identification of interacting proteins with β-catenin in driving WNT target gene transcription,^44^ to name but a few.

Therefore, we employed qPCR to detect WNT-3A induced gene transcription of *Axin2* as an orthogonal assay independent of luciferase activity in wild-type mouse embryonic fibroblasts (WT-MEFs). WT-MEFs have been shown to display a large accumulation of nuclear β-catenin upon WNT-3A treatment^45^ and *Axin2* induction,^41^ indicative of WNT/β-catenin activation. To investigate WNT-induced effects on *Axin2,* total RNA was isolated from MEFs treated with medium containing C59 (control) supplemented with 200 ng/mL WNT-3A and/or compound **3** for 24 h. We justify the use of MEFs over HEK293T cells for the qPCR analysis with a better responsiveness to WNTs on the level of *Axin2* transcription. Furthermore, it should be emphasized that Li et al. employed HEK293T cells in the TOPFlash-based SAR approach. HEK293T cells express several FZDs on the mRNA level including FZD_7_ – similar to MEFs–and thus the original assay design did not probe for FZD_7_ selective effects. In accordance with previous studies, the mRNA expression of *Axin2* increased upon treatment with 200 ng/mL WNT-3A relative to control conditions containing porcupine inhibitor, C59. Interestingly, addition of compound **3** alone had no effect on the WNT-3A-induced transcription of the *Axin2* gene (Figure 3C), which stands in stark contrast to the reported inhibitory action of compound **3**. Thus, we confirm that compound **3** acts not only as a luciferase inhibitor independent of FZD_7_ but also fails to inhibit WNT-3A-induced expression of *Axin2* as a bona fide WNT target gene in cells with a mixed expression profile of FZDs including FZD_7_.

## Discussion and Conclusions

FZD_7_ remains a widely investigated target toward drug development given its prominent role in various diseases, for example in intestinal cancer.^46^ The recent report on a series of potentially FZD_7_-targeting compounds identified through an in silico docking campaign, enabled by high resolution cryo-EM structures of FZD_7_, aimed to advance toward the goal of targeting FZD_7_ pharmacologically.^11^ However, upon further validation of the most potent compound, off-target effects were observed. This compound was derived from an accessible molecular library, which among others, contains upward of a billion compounds that are used for virtual screening. It is widely known that compound libraries contain substances that can frequently generate false hits in screening campaigns leading to a substantial waste of time and resources in both academia and industry.^47,48^ It is important to establish guidelines that can provide a cost-effective pipeline toward success. One such set of guidelines evaluates docking parameters for various targets with specific controls including identifying spectroscopic interference and subsequent test for off-target activity.^48^ Other reports include the application of chemical filters that can be employed to detect potentially problematic scaffolds such as previously reported luciferase inhibitors, which yield deceptive results with multitarget effects.^38^

In this study, we elaborate on the consequences of one such compound and pitfalls in connection with the widely used TOPFlash assay. Regarding WNT/FZD signaling, the TOPFlash readout presents a prime functional readout with scalability for high throughput screening.^20^ Nevertheless, the nature of the TOPFlash assay design as a ratiometric luciferase-based methodology demands particular discretion provided the frequency of Fluc inhibitors in compound libraries.^38^ Furthermore, compound **3** contains the problematic scaffold, 2-aminothiazole, which represents a frequent hitting scaffold in biophysical binding assays,^33^ consequently posing another complication when it comes to the use of SPR for confirmation of target binding of the compound. This class of promiscuous compounds was also identified to be photoreactive^49^ and potentially toxic.^50^ Altogether, assay-interfering compounds are ubiquitous and compromise various detection methods such as biophysical characterization or luciferase-based assay methodology. With the growing number of luciferase inhibitors, computational methods have been developed toward predicting potential luciferase inhibitors.^51^ This could represent an important early step for any compound tested regardless of its origin in virtual screening or structure-based drug design.

The confounding nature of such compounds is routinely screened against other ambiguous compounds called pan assay interference compounds (PAINS), which was introduced in 2010 to flag compounds with a high likelihood of bioassay interference.^49^ PAINS are characterized by substructural features that frequently confound screening-based drug discovery approaches. Routine screening for PAINS in connection with compound screens and SAR allows awareness of problematic chemotypes with known promiscuity, assay interference potential or fluorescence at an early stage in the hit identification, validation and optimization process (such as PrATs). A general strategy to prevent false hits can include PAINS screening as well as the incorporation of chemical filters to exclude potentially interfering scaffolds, such as those belonging to the notorious group of 2-aminothiazoles.^33^

In this study, we validated compound **3** as a potent luciferase inhibitor, which was originally identified as a surmised FZD_7_ negative allosteric modulator based on TOPFlash as the SAR-driving assay methodology. The absence of suitable counter screens and orthogonal assays for hit validation resulted in the unfortunate and false interpretation of the dramatic compound-induced reduction of the TOPFlash response as on target (FZD_7_) effect. The design of counter assays enabled us here to clearly pinpoint assay interference by compound **3**, which quenches Fluc with very high potency and does not act through FZD_7_. The key counter assay employed ΔFZD_1–10_ HEK293T cell lines and luciferase expression, whereby quantification of the WNT- and FZD-independent inhibition of Fluc activity by compound **3** was assessed. In addition, we showed that compound **3** abrogates the TOPFlash response elicited by pharmacological inhibition of GSK-3β in the complete absence of FZDs and secreted WNTs thus further emphasizing that compound **3** targets Fluc and not FZD_7_. Lastly, and most importantly, WNT-3A-induced conformational rearrangements in a FZD_7_-DEP-Clamp biosensor and transcription of a bona fide WNT/β-catenin-target gene, *Axin2,* were unaffected by compound **3**. In this regard, the lack of an effect of compound **3** on WNT-3A-induced and FZD-mediated effects aligns with the artifact nature identified initially by the inhibition of a WNT-3A-induced TOPFlash signal. The FZD_7_-DEP-Clamp sensor and detection of mRNA by qPCR served here as a Fluc-independent orthogonal assays reporting a WNT-induced and FZD-mediated response.

This study serves as a cautionary tale for rational drug design toward FZDs, and we want to underline that–irrespective of the drug target in focus–assay design and inclusion of suitable counter assay methodology is essential for drug screening, SAR, and hit validation. Despite this setback, improved assay methodologies and better understanding of FZD activation mechanisms should guide the continued quest for potent and potentially paralog selective FZD-targeting small molecules.

## Experimental Section

### Chemical Materials

Solvent and reagents are obtained from commercial sources and used as received. ^1^H NMR and ^13^C NMR spectra are recorded on Bruker AVANCE 400 (^1^H: 400 MHz; ^13^C: 101 MHz) spectrometer. For ^1^H NMR spectra, solvents are referenced to the solvent peak (CD_3_)_2_SO (2.50 ppm) and chemical shifts are reported in parts per million (ppm). The following abbreviations are used for the description of signals: s (singlet) and m (multiplet). ^13^C NMR (DEPTQ) spectra are recorded in (CD_3_)_2_SO using (CD_3_)_2_SO (39.52 ppm) as standard. Chemical shifts are given in parts per million (ppm). Mass detection was performed on a BRUKER amazon SL mass spectrometer using electron spray ionization (ESI). RP- HPLC purity analyses were performed on an Agilent 1200 series with an Agilent Zorbax XDB-C8 (4.6 × 150 mm, 5 μm) column using a DAD-detector for UV-analyses with two different systems. System 1: methanol/0.1% aq. HCOOH, 10% for 3 min, 10% - 95% in 18 min, 95% for 4 min, 95% - 10% in 2 min, 10% for 3 min. System 2: acetonitrile/0.1% aq. trifluoroacetic acid, 10% for 3 min, 10% - 95% in 18 min, 95% for 4 min, 95% - 10% in 2 min, 10% for 3 min. All compounds produced are >95% pure by HPLC (Figure S2).

### Chemical Synthesis

#### 2-Chloro-*N*-(4-phenylthiazol-2-yl)acetamide (Compound **1**)

4-Phenylthiazol-2-amine (200 mg, 1.13 mmol) and DIPEA (250 μL, 1.48 mmol, 1.3 equiv) were solved in dry dichloromethane (5 mL) at 0 °C. Chloroacetyl chloride (100 μL, 1.25 mmol, 1.1 equiv) was slowly added and the reaction mixture was stirred overnight at ambient temperature. After completion, the solvent was removed under reduced pressure and the resulting resin was quenched by the addition of aqueous saturated NaHCO_3_. The solution was extracted with EtOAc (3 × 10 mL), and the combined organic layers were dried over Na_2_SO_4_ and concentrated with a rotary evaporator. Purification by flash-chromatography on silica gel (isohexane/ethyl acetate, 90:10 to 80:20) afforded compound **1** (226 mg, 892 μmol, 79%) as white precipitation.

^1^H NMR (400 MHz, DMSO-*d*_6_): δ 12.66 (s, 1H), 7.96–7.86 (m, 2H), 7.69 (s, 1H), 7.48–7.40 (m, 2H), 7.36–7.29 (m, 1H), 4.42 (s, 2H). ^13^C NMR (101 MHz, DMSO-*d*_6_): δ 171.4, 165.2, 149.1, 134.3, 128.8, 128.8, 128.0, 127.9, 125.7, 108.7, 42.4. ESI-MS *m*/*z* 253.0 [M + H]^+^.

#### 2-(4-Aminophenyl)thio)-*N*-(4-phenylthiazol-2-yl)acetamide (Compound **2**)

Compound **1** (113 mg, 446 μmol) was solved in dry dimethylformamide (5 mL). K_2_CO_3_ (185 mg, 1.34 mmol, 3.0 equiv) and 4-aminothiophenol (67.0 mg, 535 μmol, 1.2 equiv) were added and stirred at ambient temperature for 5 h. Aqueous saturated NaHCO_3_ was added and the reaction mixture was extracted with EtOAc (3 × 10 mL). The combined organic layers were washed with aqueous saturated NaCl (3 × 50 mL), dried over Na_2_SO_4_ and concentrated under reduced pressure. After purification by flash-chromatography on silica gel (isohexane/ethyl acetate, 80:20 to 50:50) compound **2** (138 mg, 405 μmol, 91%) was obtained as white precipitation. ESI-MS *m*/*z* 342.0 [M + H]^+^.

#### 2,4-Dimethyl-*N*-(4-((2-oxo-2-((4-phenylthiazol-2-yl)amino)ethyl)thio)phenyl)benzamide (Compound **3**)

2,4-Dimethylbenzoeacid (37.0 mg, 243 μmol, 1.2 equiv) and HATU (173 mg, 460 μmol, 2.0 equiv) were solved in DMF (2.80 mL). DIPEA (700 μL) was added and the reaction mixture was stirred for 30 min at ambient temperature. A solution of compound 2 (69.0 mg, 202 μmol) in DMF (4 mL) was added to the reaction mixture and stirred for 12 h at 60 °C. After completion, the solvent was removed under reduced pressure and the resulting resin was quenched with aqueous saturated NaHCO_3_. The mixture was extracted with EtOAc (3 × 20 mL) and the combined organic layers were dried over Na_2_SO_4_ and concentrated with a rotary evaporator. The residue was purified by flash-chromatography on silica gel (CH_2_Cl_2_/ethanol 99:1) and further purified by preparative HPLC using a solvent system of acetonitrile/0.1% aq. HCOOH, 30% - 45% in 3 min, 45% - 55% in 11 min, 55% - 95% in 2 min, 95% for 2 min, 95% - 30% in 2 min, 30% for 3 min, flow rate of 20 mL/min, *t*_R_ = 10.1 min (λ = 254 nm), column: Agilent Zorbax XDB-C8 21.2 × 150 mm, 5 μm. The preparative HPLC was performed on an Agilent 1260 Infinity System equipped with a MWD-detector. Compound **3** (75.7 mg, 159.8 μmol, 79%) was obtained as a white solid.

^1^H NMR (400 MHz, DMSO-*d*_6_): δ 12.48 (s, 1H), 10.27 (s, 1H), 7.91–7.87 (m, 2H), 7.76–7.65 (m, 2H), 7.62 (s, 1H), 7.47–7.25 (m, 6H), 7.15–7.03 (m, 2H), 3.90 (s, 2H), 2.32 (m, 6H). ^13^C NMR (151 MHz, DMSO-*d*_6_): δ 167.9, 167.8, 148.8, 139.3, 138.4, 135.4, 134.3, 134.1, 131.2, 130.4, 129.6, 128.7, 127.8, 127.4, 126.1, 125.6, 120.1, 108.1, 37.5, 20.8, 19.4. ESI-MS *m*/*z* 474.4 [M + H]^+^. RP-HPLC (System 1): _R_ = 23.7 min, purity: 97.1% (254 nm). RP-HPLC (System 2): *t*_R_ = 20.8 min, purity: 98.6% (254 nm).

### Cell Culture and Ligands

ΔFZD_1–10_ HEK293T and mouse embryonic fibroblast (MEFs) were cultured in Dulbecco’s modified Eagle’s medium (DMEM) supplemented with 10% fetal bovine serum, 1% penicillin/streptomycin, and 1% l-glutamine (Thermo Fisher Scientific) in a humidified CO_2_ incubator at 37 °C. All cell culture plastics were from Sarstedt unless otherwise specified. Recombinant untagged WNT-3A was from R&D Systems/Biotechne (nos 5036-WN). WNT-3A was dissolved in filter-sterilized 0.1% BSA in HBSS (HyClone) along with the vehicle control. C59 (2-[4-(2-methylpyridin-4-yl)phenyl]-*N*-[4-(pyridin-3-yl)phenyl]acetamide; (Abcam #ab142216) was stored as 5 mM solution in aliquots in dimethyl sulfoxide (DMSO; at −20 °C) and used to inhibit porcupine to abrogate endogenous secretion of WNTs. For stimulation, recombinant WNT-3A (R&D Systems/Biotechne #5036-WN) was used. Transfections were performed using PEI (Alfa Aesar, linear, MW 25,000, stock solution: 1 mg/mL; PEI (μL): DNA (μg) ratio 5:1). CHIR99021 is from Cayman Chemical Company (Item 13122) and resuspended in DMSO at a stock concentration of 10 mM. The absence of mycoplasma contamination was routinely confirmed by polymerase chain reaction using 5′-GGCGAATGGGTGAGTAACACG-3′ (forward) and 5′-CGGATAACGCTTGCGACTATG-3′ (reverse) primers detecting 16S rRNA of mycoplasma in the medium after 2–3 days of cell exposure.

### Molecular Cloning

Plasmids were generated using Gibson Assembly. Generation of the HiBiT-FZD_7_ construct was described previously.^52^ The plasmid used for constitutive expression of the firefly luciferase (Fluc) under control of a CMV promoter was generated via Gibson Assembly. To do so, the sequence for DEP-Venus in pVenus-N1 background^40^ was replaced with the sequence for Fluc, which was amplified from the Super8X TOPFlash reporter gene plasmid (Addgene #12456). To generate the TK-Nluc plasmid, the Renilla luciferase (pRL) in pRL-TK (Promega) was exchanged with the sequence for Nluc, which was amplified from HA-FZD_5_-Nluc^53^ (via Gibson Assembly). All sequences were verified by Sanger Sequencing (Eurofins Genomics).

### TCF/LEF Luciferase Reporter Assay (TOPFlash)

HEK293T ΔFZDs_1–10_ were transfected in suspension at a concentration of 450,000 cells/mL with 200 ng of HiBiT-FZD_7_, 250 ng of the M50 Super 8x TOPFlash reporter (Addgene #12456) and 50 ng of Renilla luciferase control plasmid (pRL-TK, Promega), which contains a herpes simplex virus thymidine kinase (HSV-TK) promoter. Empty pcDNA3.1 was used to adjust the transfected DNA amount to 1 μg per mL cell suspension. 100 μL of cells (45,000 cells/well) were seeded on a white-bottomed, PDL-precoated, 96-well microtiter plate (Thermo Fisher Scientific). After 24 h, the medium was removed and serum-free DMEM medium containing 300 ng/mL WNT-3A or vehicle supplemented with 10 nM of the porcupine inhibitor C59 was added. Ten μM of compound **3** or DMSO (control) was added after. After 24 h of stimulation, the Dual Luciferase Assay Kit (Promega, #E1910) was used to prepare the cells. The cells were lysed in 20 μL of 1× passive lysis buffer for 15 min at room temperature with gentle shaking. Subsequently, 20 μL of LARII reagent were added, and β-catenin-dependent Fluc bioluminiscence was read on a Spark multimode microplate reader (Tecan, 550–620 nm, integration time: 2 s). Lastly 20 μL of 1× Stop-and-Glo reagent were added per well, and Rluc bioluminiscence was read (445–530 nm, integration time: 2 s).

### Fluc Bioluminescence Assay

HEK293T ΔFZDs_1–10_ were prepared as before by transfecting them with 10 ng of Fluc construct (adjusted to 1 μg per mL cell suspension with pcDNA3.1). After 24 h, the medium was removed and serum-free DMEM medium containing 10 nM C59 and a concentration range of compound **3** (0.1–1000 nM) was added. Twenty-four hours after addition of compound **3**, medium was removed, washed 1× with HBSS and 20 μL of 1× passive lysis buffer were added for 15 min at RT with gentle shaking. Subsequently, β-catenin-dependent Fluc bioluminescence was read on a Spark multimode microplate reader after addition of LARII reagent as described above (Tecan, 550–620 nm, integration time: 2 s).

### Rluc/Nluc Response Assay

HEK293T ΔFZDs_1–10_ were prepared as before by transfecting them with 50 ng of Renilla luciferase controlconstruct (pRL-TK) or 20 ng of Nluc control (TK-Nluc) and adjusted to 1 μg per mL cell suspension with pcDNA3.1. After 24 h, the medium was removed and serum-free DMEM medium containing 10 nM C59 and a concentration range of compound **3** (0.1–1000 nM) was added. Twenty-four h after addition of compound **3**, medium was removed, washed 1× with HBSS. For Rluc measurements, 20 μL of 1× Stop-and-Glo reagent were added per well, and Rluc bioluminescence was read (445–530 nm, integration time: 2 s). For Nluc measurements, 90 μL of HBSS was added followed by 10 μL of furimazine (Nano-Glo Substrate, Promega, #N1572, prediluted 1:1000 in HBSS). The plate was incubated in the dark for 6 min and luminescence was read (445–485 nm, integration time: 2 s).

### FZD-DEP-Clamp Experiments

For transient transfection, HEK293T ΔFZDs_1–10_ were prepared as before and transfected with 20 ng of FZD_7_-DEP-Clamp construct and adjusted to 1 μg per mL cell suspension with pcDNA3.1. After 48 h, cells were washed once with HBSS and kept in 70 μL of HBSS (for determination of basal BRET ratios) or 0.1% BSA/HBSS (for ligand stimulation). Compound **3** (final concentration 10 μM) or DMSO were prepared in HBSS and 10 μL were added to the plate. The substrate, furimazine (NanoBRET Nano-Glo Substrate, Promega, #N1572, prediluted 1:100 in 0.1% BSA/HBSS) was added to the cells and incubated for 10 min inside the plate reader (prewarmed to 37 °C). Three consecutive BRET reads were performed (basal BRET), and then 10 μL of vehicle or 1 μg/mL WNT-3A (in 0.1% BSA/HBSS) were added. Following stimulation, the BRET measurement was read every 2 min for 1 h (Nluc bioluminescence 445 nm-485 nm, mVenus emission 520–560 nm, integration time: 100 ms).

### RNA Extraction and Quantitative PCR (qPCR)

Mouse embryonic fibroblasts (MEFs) were seeded in a 6-well plate at a concentration of 140,000 cells/mL. After 24 h, medium was replaced with serum-free DMEM supplemented with C59 and either treated with WNT-3A (200 ng/uL), compound **3** (10 μM), or both and incubated for 24 h. Total cellular RNA was isolated using The RNeasy Plus Mini Kit (Qiagen). The quality and concentration of RNA were assessed by a spectrophotometer (NanoDrop) yielding 260/280 absorbance ratio values between 1.9 and 2.1 indicating RNA samples were of good purity. Reverse transcription was undertaken on RNA (500 ng) using SuperScript IV Reverse Transcriptase (Thermo Scientific) in a reaction volume of 10 μL. The resulting cDNA was used for qPCR with gene-specific primers for mouse *Axin2 (*adapted from GenBank Accession code NM_015732). Standard primers for Actin-β were used as the internal reference. Master mixes were prepared using the iTaq Universal SYBR Green Supermix (BioRad) and comparative quantification was conducted. Relative gene expression of *Axin2* was calculated using the 2^–ΔΔCt^ method.^54^ Data are presented as fold change compared to the baseline conditions (in the presence of C59). The internal reference primers are reported as follows: actin-β forward, “CGCAGCCACTGTCGAGT “, actin-β reverse “CCCACGATGGAGGGGAATAC “. The primer for *Axin2* used is as follows: *Axin2* forward, “AACCTATGCCCGTTTCCTCTA “, *Axin2* reverse, “GAGTGTAAAGACTTGGTCCACC “.

### Data and Statistical Analysis

Raw data was obtained from the plate reader as a Microsoft Excel spreadsheet (.xlsx format) and analyzed using GraphPad Prism. TOPFlash ratios were determined by dividing the Fluc emission (β-catenin-dependent transcriptional activity) by the Rluc emission (indicator for transfection efficiency). TOPFlash ratios between the different conditions were tested for statistical significance using one-way analysis of variance (ANOVA) followed by Tukey’s posthoc analysis (*p* < 0.05). The control assays with Fluc and Rluc were plotted as raw luciferase counts (RFU) obtained from the plate reader. For ligand stimulation experiments with the FZD_7_-DEP-Clamp sensor, the average of the first three reads (basal BRET) was subtracted for every well (baseline-corrected BRET) to adjust for variation between wells. Then, the average of the baseline-corrected BRET ratios from vehicle-stimulated wells was subtracted from the baseline-corrected BRET ratios of ligand-stimulated wells to infer on the ligand effect (ΔBRET). The ΔBRET values were calculated for each independent experiment using GraphPad Prism and then averaged to generate the figure. The control assay with Nluc is plotted as normalized luciferase counts (RFU) where absence of compound 3 is equal to 1. Differences among treatment groups for qPCR were analyzed using the 2^–ΔΔCt^ method and by one-way ANOVA (Dunnett’s multiple comparison test) with *p* < 0.05 being considered significant. The software package, Prism 6.0 (GraphPad Software, La Jolla, CA, U.S.A) was used to perform all analyses.

Details about the synthesis of compounds (Figure S1) with HPLC traces and ^1^H NMR (Figure S2) used in the study are provided.

## Supporting Information Available

Chemical synthesis, ^1^H NMR, and HPLC of compound **3** (PDF)
Molecular formula strings of representative compounds (CSV)

## Abbreviations

C59: 2-[4-(2-methylpyridin-4-yl)phenyl]-*N*-[4-(pyridin-3-yl)phenyl]acetamide
CRC: concentration response curve
DVL: Dishevelled
ESI: electron spray ionization
Fluc: Firefly luciferase
GPCR: G protein-coupled receptor
HPLC: high-pressure liquid chromatography
Nluc: Nanoluciferase
NMR: nuclear magnetic resonance
qPCR: quantitative PCR
Rluc: Renilla luciferase
SAR: structure–activity relationship
SBVS: structure-based virtual screening
TCF/LEF: T-cell factor/lymphoid enhancer binding factor
WNT: Wingless/Int1
