# Supplementary material for: A Putative Frizzled 7-Targeting Compound Acts as a Firefly Luciferase Inhibitor
Source: J Med Chem. 2024 Dec 13;67(24):22332–41. doi: 10.1021/acs.jmedchem.4c02766 (PMC11684006; doi:10.1021/acs.jmedchem.4c02766)
Supplement: Supplementary file 1 — jm4c02766_si_001.pdf [file jm4c02766_si_001.pdf]

Supporting Information

A putative Frizzled 7-targeting compound acts as a Firefly luciferase inhibitor

Julia Kinsolving[1], Lukas Grätz[1], Jan Voss[1], Bente Löw[2], Emily Shorter[3], Baptiste Jude[3,4], Johanna T Lanner[3], Stefan Löber[2], Peter Gmeiner[2], Gunnar Schulte[1,*]

[1] Section of Receptor Biology & Signaling, Dept. Physiology & Pharmacology, Karolinska Institutet, S-171 77 Stockholm, Sweden

[2] Department of Chemistry and Pharmacy, Friedrich-Alexander-Universität, 91058 Erlangen, Germany

[3] Molecular Muscle Physiology and Pathophysiology, Dept. Physiology & Pharmacology, Karolinska Institutet, S-171 77 Stockholm, Sweden

[4]Division of Pediatric Neurology, Dept. Of Women's and Children's Health, Karolinska Instituett, S-171 77 Stockholm, Sweden

*****corresponding author email: gunnar.schulte@ki.se

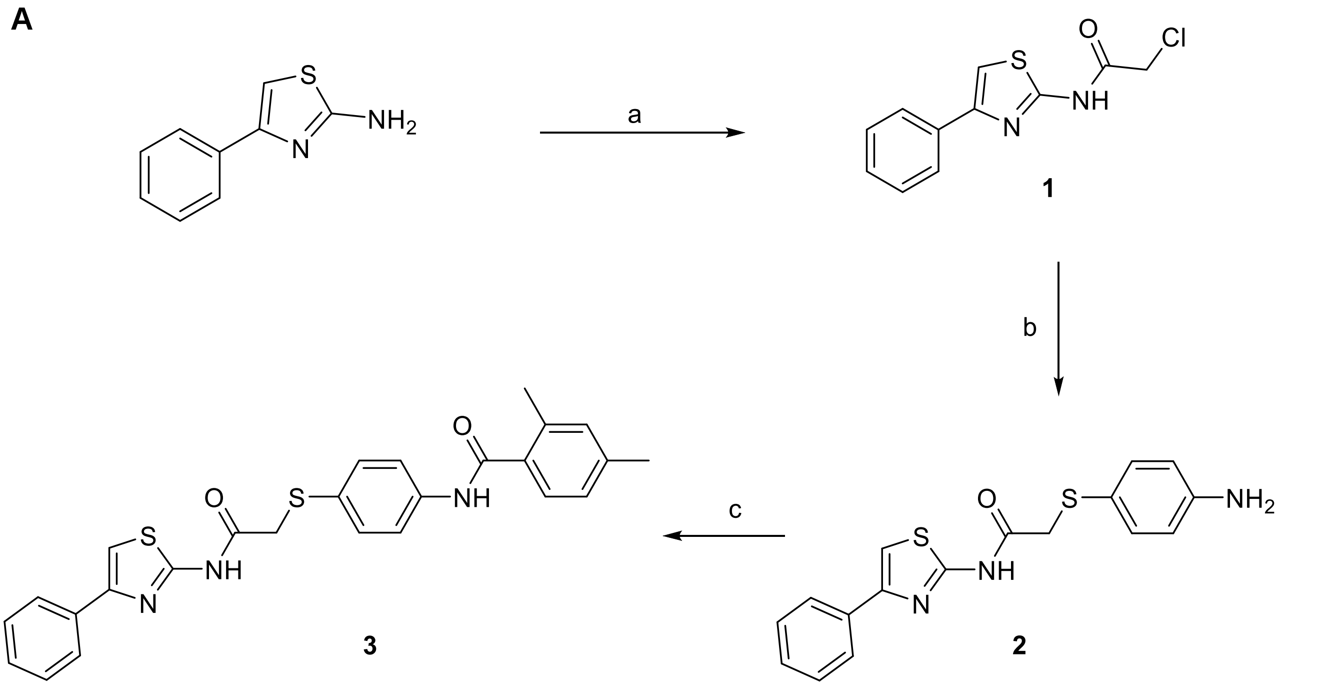

Fig. S1: **Synthesis of compound 3**

(**A**) Scheme of synthesis of compound **3**. Reagents and conditions: (a) Chloracetyl chloride, DIPEA, DCM, 0°C - r.t., 12 h, yield 79%; (b) 4-aminothiophenol, K_2CO_3, DMF, r.t., 6 h, yield 91%; (c) 2,4-methylbenzoeacid, HATU, DIPEA, DMF, r.t. - 60°C, 12 h, yield 79%. Compounds are reported as follows *2-Chloro-N-(4-phenylthiazol-2-yl)acetamide (compound **1**); 2-(4-Aminophenyl)thio)-N-(4-phenylthiazol-2-yl)acetamide (compound **2**); 2,4-Dimethyl-N-(4-((2-oxo-2-((4-phenylthiazol-2-yl)amino)ethyl)thio)phenyl)benzamide (compound **3**)*

A

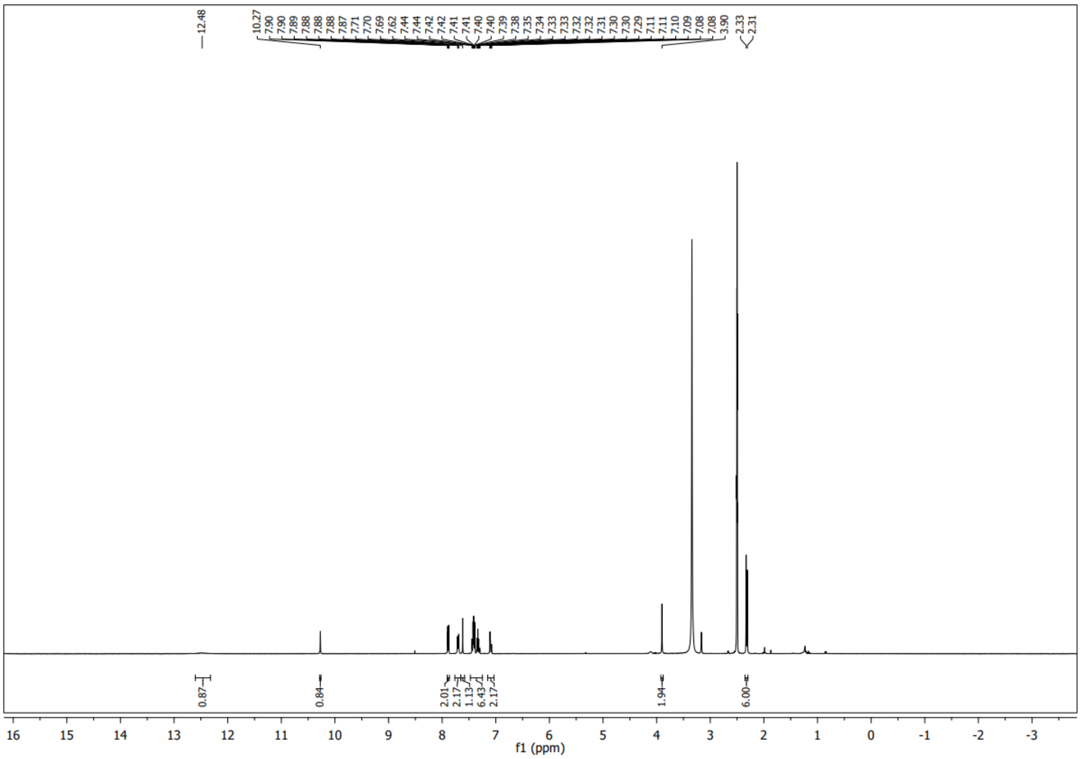

B System 1:

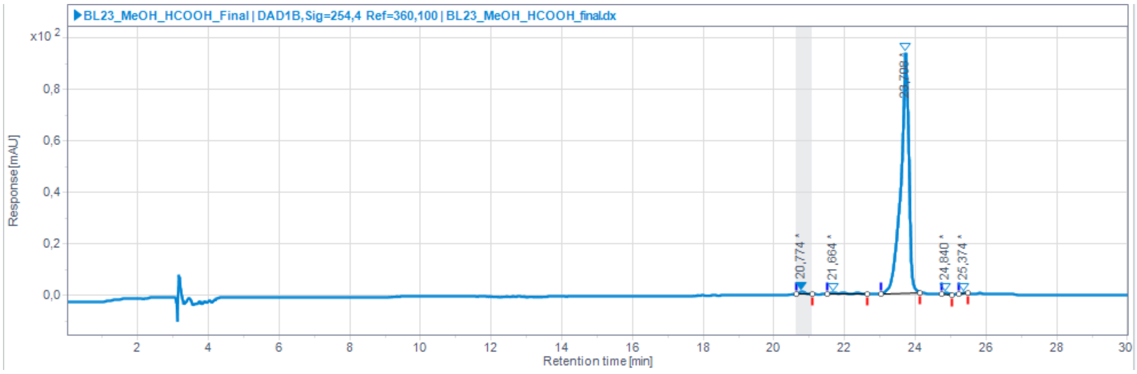

System 2:

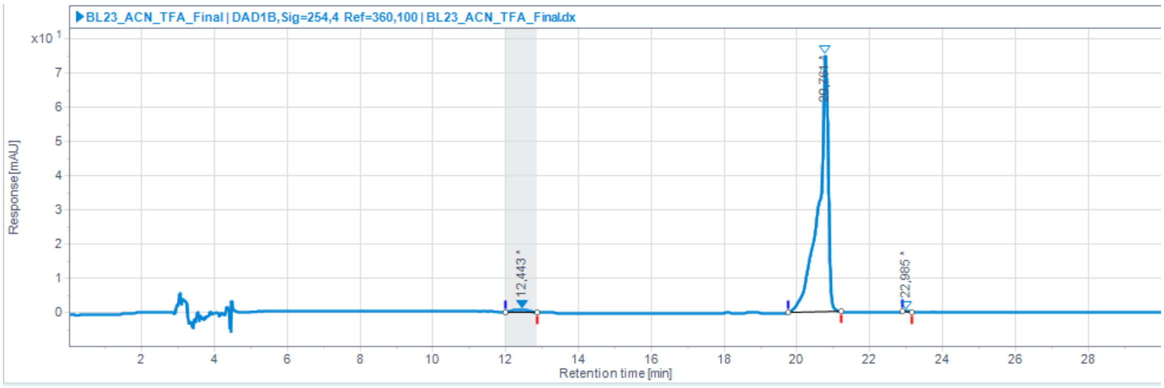

Fig. S2: **^{1}H-NMR & HPLC**

(**A**) Compound **3**: ^{1}H NMR (400 MHz, DMSO-d_6): δ 12.48 (s, 1H), 10.27 (s, 1H), 7.91 – 7.87 (m, 2H), 7.76 – 7.65 (m, 2H), 7.62 (s, 1H), 7.47 – 7.25 (m, 6H), 7.15 – 7.03 (m, 2H), 3.90 (s, 2H), 2.32 (m, 6H). ^{13}C NMR (151 MHz, DMSO-d_6): δ 167.9, 167.8, 148.8, 139.3, 138.4, 135.4, 134.3, 134.1, 131.2, 130.4, 129.6, 128.7, 127.8, 127.4, 126.1, 125.6, 120.1, 108.1, 37.5, 20.8, 19.4. ESI-MS m/z 474.4 [M+H]$^{+}$. (**B**) RP-HPLC (System 1): $_{R}$ = 23.7 min, purity: 97.1% (254 nm). RP-HPLC (System 2): t_R = 20.8 min, purity: 98.6% (254 nm)
